# Manufacture of Binary Nanofeatured Polymeric Films Using Nanosphere Lithography and Ultraviolet Roller Imprinting

**DOI:** 10.3390/ma14071669

**Published:** 2021-03-29

**Authors:** Demei Lee, Ming-Yi Hsu, Ya-Ling Tang, Shih-Jung Liu

**Affiliations:** 1 Department of Mechanical Engineering, Chang Gung University, Taoyuan 33302, Taiwan; dmlee@mail.cgu.edu.tw (D.L.); m7259@cgmh.org.tw (M.-Y.H.); vellick27candy@yahoo.com.tw (Y.-L.T.); 2 Department of Medical Imaging and Intervention, Chang Gung Memorial Hospital-Linkou, Taoyuan 33305, Taiwan; 3 Department of Diagnostic Radiology, Chang Gung Memorial Hospital-Keelung, Keelung 20401, Taiwan; 4 Bone and Joint Research Center, Department of Orthopedic Surgery, Chang Gung Memorial Hospital-Linkou, Taoyuan 33305, Taiwan

**Keywords:** nanosphere lithography, roller imprinting, binary nanofeatured film

## Abstract

This paper describes the manufacture of binary nanostructured films utilizing nanosphere lithography and ultraviolet (UV) roller imprinting. To manufacture the binary nanofeatured template, polystyrene nanocolloids of two distinct dimensions (900 and 300 nm) were primarily self-assembly spun coated on a silicon substrate. A roller imprinting facility equipped with polydimethylsiloxane molds and ultraviolet radiation was employed. During the imprinting procedure, the roller was steered by a motor and compressed the ultraviolet-curable polymeric layer against the glass substrate, where the nanofeatured layer was cured by the UV light source. Binary nanofeatured films were thus obtained. The influence of distinct processing variables on the imprinting of nanofeatured films was investigated. The empirical data suggested that with appropriate processing conditions, binary nanofeatured plastic films can be satisfactorily manufactured. It also demonstrated that roller imprinting combined with ultraviolet radiation can offer an easy yet effective method to prepare binary nanofeatured films, with a miniatured processing time and enhanced part quality.

## 1. Introduction

Subsiding the sizes of a substance to the nanoscale generally leads to the variation of physical/chemical properties. Nanotechnology permits the achievement of novel materials/devices with essential structural element in nanoscale and is assessed via managing at either the atomic, molecular, or supramolecular level. The unique characteristic of binary nanostructure (or micro/nanostructure) [1] has been used in photosensors (with modification of optical properties in absorption, reflection, and color), promoted Raman imaging [2], and enhanced energy storage and transformation efficiency in photovoltaics [3,4]. The structure has also been employed for varying material surface’s wettability, thus providing advantages in applications comprising self-cleaning, anti-icing, fluidic control and drag reduction [5,6,7,8]. Meanwhile, material surface with managed topographic features at the micro and nanoscales has been demonstrated to influence the entire cell behavior as well as the ultimate cell/material integration [9,10]. Binary nanofeatured surface can also be used to direct differentiation into a specific cell lineage in the nanoscale circumstance [11,12]. The development of manufacturing methods for binary nanofeatured surface is thus highly desired.

Owing to its advantages, the roller imprinting [13] has been swiftly exploited during the past decade as a favorable option to traditional nanofabriction methods to satisfy the needs resulting from the new progresses in the semiconductor and flexible electronics industries, etc. It is also the most demanding technology, owing to the high yield for industrial fabrications. During the imprinting process, a pre-manufactured mold holding an opposite of the needed features is compressed against a resist-coated substrate to duplicate the features by deformation. Various duplications can be completed from one sole pre-manufactured mold employing this scheme. Additionally, roller ultraviolet (UV) imprinting technology [14], due to its advantages of low-cost, high-yield, and vast-area imitating, has received increasing attentions from both academia and industries for the successive manufacture of nanostructures such as optical lithography, deposition, and etching. The innovative UV roller imprinting technique allows the patterning of high-quality structured layers on glass/glass-based devices at lowest costs. With regard to the molding performance, distinct 2D/3D features with dimensions spanning from few micrometers to sub-nanometers have been successfully achieved [15].

Nanospheres lithography [16,17] is a fabrication process with reference to the self-assembly of nanocolloids. A lithography mask is first prepared by submerging the substrate in the nanocolloids suspension. After the vaporization of solvent, a self-assembled monolayer is created on the substrate surface. This is followed by the staking of the aspired substance onto the lithography mask. After the removal of the template, a periodic array of nanocolloids is obtained. The process has received increasing attention in recent years, mainly owing to the feasibility of producing regular patterns onto large area with rational cost [18].

In this study, we detailed the manufacture of binary nanofeatured films via a soft-mold roller imprinting device with UV radiation capability. Binary nanofeatured template was first prepared by self-assembling polystyrene nanocolloids of two distinct dimensions (i.e., 900 and 300 nm) on silicon substrates. The soft mold [19] was acquired by pouring the polydimethylsiloxane (PDMS) solution onto the template so as to obtain a binary nano-cavity array for roller imprinting. In imprinting, the roller rotates and presses the UV-curable polymeric layer onto the glass substrate. Once cured, binary nanofeatured polymeric films were acquired and characterized. The impact of different variables on the imprinting of nanostructured films was also explored.

## 2. Materials and Method

### 2.1. Materials

Colloidal nanospheres of polystyrene (PS) of 900 nm/300 nm were provided by micro-Particles GmbH (Berlin-Adlershof, Germany). Other materials employed for the experiments included surfactant Triton X-100 and ethanol acquired from Sigma-Aldrich (St. Louis, Mo, USA), polydimethylsiloxane (PDMS) SYLGARD 184 pre-polymer mixture and cross-linker from Dow Corning (Elizabethtown, KY, USA), DS-UPS-Aw dispersant provided by Golden Innovation (Taipei, Taiwan), FL171-10 ultraviolet curable epoxy resin, with a refractive index 1.45 at 365 nm wavelength and a viscosity of 320–470 cps at 25 °C, from Everwide Chem. (Taipei, Taiwan), and hexane purchased from JT-Baker (Phillipsburg, NJ, USA).

### 2.2. Prepare the Nanofeatured Template

**Figure 1:**
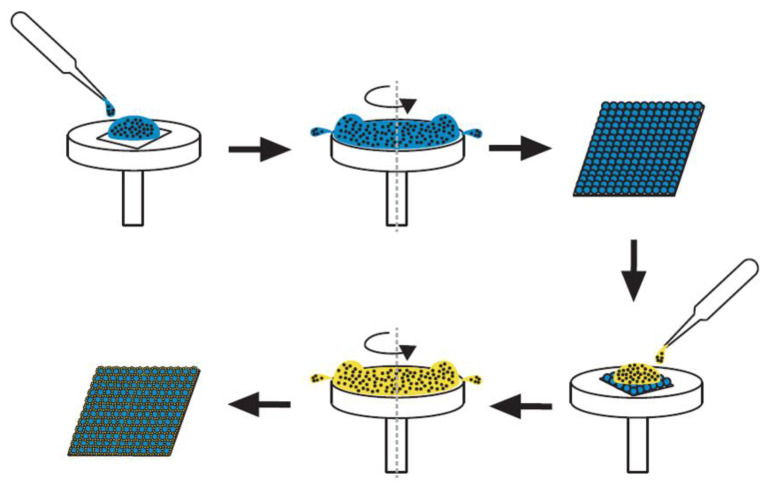
The schematic experimental process for assembly of binary nanosphere arrays.

Figure 1 shows schematically the procedure for assembly of binary nanosphere arrays. PS nanospheres of 300 nm were first mixed with the surfactant at a ratio of 0.7:0.5 (*v*/*v*), while the colloidal nanospheres of 900 nm were compounded with the surfactant at a ratio of 0.3:0.5 (*v*/*v*). The solution of 900 nm nanospheres were spun coated onto the substrate, using a distilled (DI) water:ethanol ratio of 1:1, a surfactant:PS sphere ratio of 1:2, and 5% wt of dispersant. Three stages of spin speed (spin time) were employed, i.e., 500 rpm for 30 s, accompanied by 1500 rpm for 30 s, and 2000 rpm for 60 s. After the coating of 900 nm spheres, the solution of 300 nm nanospheres was then spun coated employing a DI water:ethanol ratio of 1:1, a surfactant:PS sphere ratio of 1:2, 10% wt of dispersant, and spin speed (spin time) of 3000 rpm (30 s). Table 1 lists the parameters used in the sequential spin coating process.

### 2.3. Preparation of Soft Mold

**Figure 2:**
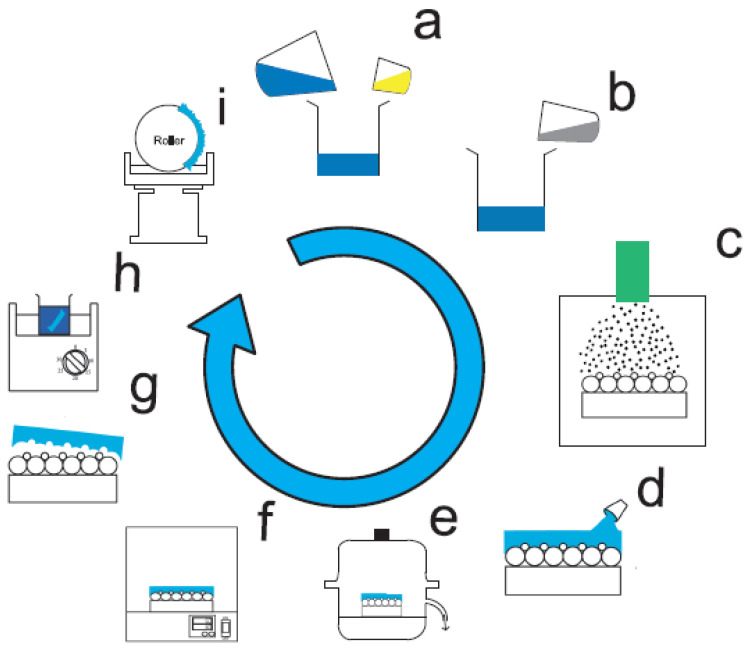
Schematic prepation of soft mold.

Polydimethylsiloxane (PDMS) pre-polymer solution was first mixed with the cross-linker (Figure 2a), followed by the addition of hexane (Figure 2b). Assembled binary array was the nanocolloid-patterned substrate, which was plasma sputtered (Figure 2c) via a sputtering device (PDC-001, Harrick Plasma, Ithaca, NY, USA) and Argon gas. The sputtering time was 5 min. The soft mold was prepared by casting the PDMS pre-polymer mixture over the binary nanosphere array that acts as a template (Figure 2d). After being placed in a vacuum chamber for 5 min (Figure 2e), the PDMS mold was put in an isothermal oven at 60 °C for 3 h (Figure 2f). The soft mold was trimmed from the template post-curing (Figure 2g), and was placed in acetonitrile for ultrasonication for 30 min to eliminate remaining PS nanospheres on the mold (Figure 2h). A PDMS mold with nanocavities was thus obtained.

### 2.4. Roller Imprinting of Nanofeatured Films

Imprinting tests were completed utilizing a lab-developed UV roller imprinting apparatus (Figure 2i) [20], which includes a UV light source, a roller PDMS mold, a speed-controllable motor-steered table, and a reservoir that holds the light curable polymer. The UV source’s utmost power (ByWell Mater., New Taipei City, Taiwan) is 3900 mW/cm^2^, possessing a 365 nm wavelength. Two distinct amounts of light radiations, 530 and 3900 mW/cm^2^, were utilized. The travel rate of the table was set at 5.2, 13.1, or 20.9 mm/s. The imprinting pressures were modified by altering the roller/glass substrate clearance, which were adjusted via two Z-stages situated above the table. Three clearances, −200, 0 and 100 μm, were adopted for the imprinting procedure. The negative clearance implies intervention of the roller and glass substrate.

During roller imprinting, the UV-curable resin was initially retained in the reservoir. The PDMS mold contacted the resin once the roller stamp rotated. The photopolymer mixture was compressed against the nanocavities on the PDMS roller. Upon the roller contacting the glass substrate, polymeric films with binary nanostructures were created on the glass substrate following the UV irradiation. Once peeled off from the substrate, nanofeatured film was acquired.

## 3. Results and Discussion

**Figure 3:**
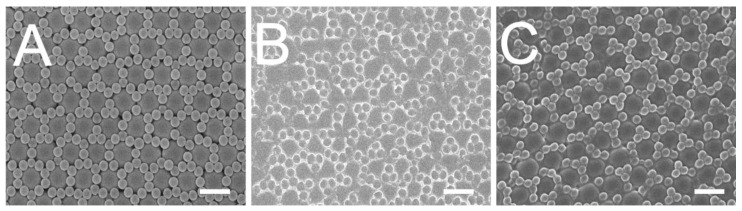
SEM images of (**A**) assembled 300 nm/900 nm binary array, (**B**) replicated soft mold, (**C**) imprinted nanofeatured film. (Scale bar: 1 μm).

**Figure 4:**
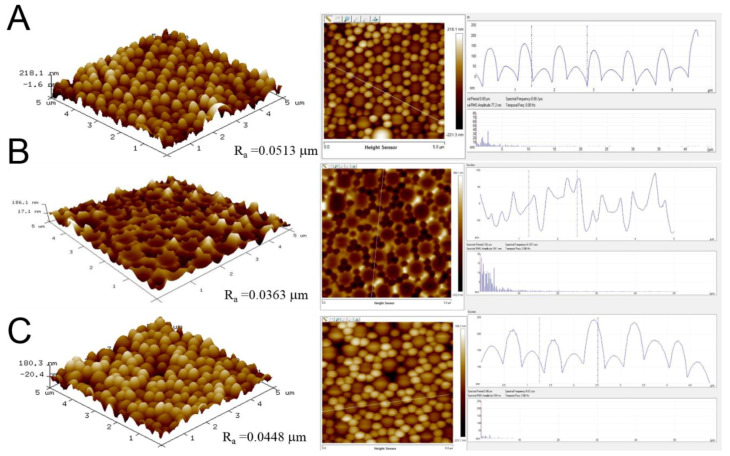
AFM surface morphologies of (**A**) assembled binary nanosphere array, (**B**) replicated soft mold, (**C**) imprinted nanofeatured film.

Figure 3A,B show the scanning electron microscopy (SEM) images of self-assembled nanosphere array and replicated soft mold, respectively. The arrays were analyzed utilizing an atomic force microscope (AFM). Figure 4A,B display the evaluated imaged of the self-assembled binary 900 nm/300 nm nanocolloid array and replicated soft mold, respectively. The average surface roughnesses (Ra) thus obtained were 51.3 nm and 36.3 nm, respectively. The experimental data show that the spin coating technology can satisfactorily self-assemble the 900 nm/300 nm nanocolloids on the substrate with consistent distributions. Furthermore, the binary nanostructured arrays were effectively duplicated onto the PDMS mold with well distributions.

**Figure 5:**
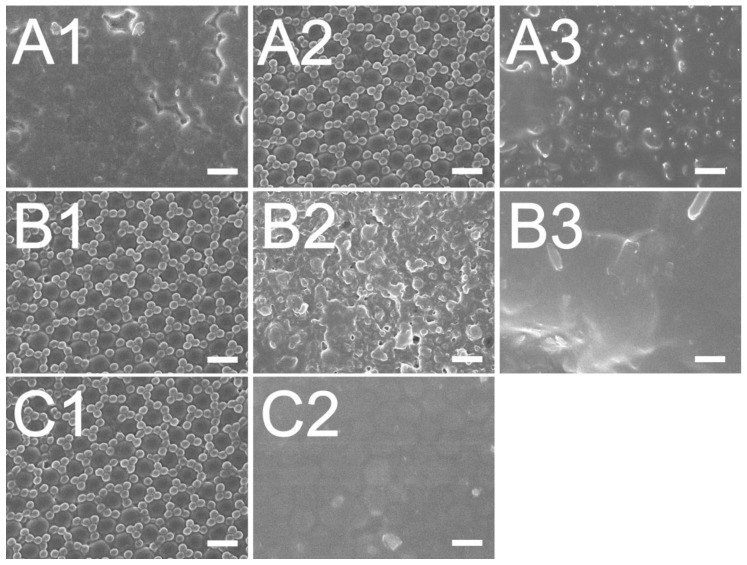
Influence of (**A**) distance between roller stamp and glass substrate; (**A1**): +100 μm, (**A2**): 0 μm, (**A3**): −200 μm, (**B**) rolling speed; (**B1**): 5.23 mm/s, (**B2**): 13.08 mm/s, (**B3**): 20.93 mm/s, (**C**) UV amount; (**C1**):3900 mW/cm^2^, (**C2**): 530 mW/cm^2^, on the manufacture of 300 nm/900 nm binary nanostructured films. (Scale bar: 1 μm).

The imprinting device equipped with the soft mold was used to mold the binary nanofeatured films. The impact of the clearance between the roller and the glass substrate was investigated. The empirical outcome (Figure 5A) indicates that a clearance of 0 μm imprinted films possessed the utmost superior duplicability. The conformation of polymeric film to the soft mold’s nanofeatures is the main concern for roller imprinted films. During the imprinting procedure, a pressure is imposed on the soft mold on the roller to steer the photopolymer solution to flow into the nanocavities. The applied pressure can be enhanced by reducing the clearance between the roller and the substrate. When the pressure is too low, not enough polymer liquid is compressed into the nanocavities. Imprinted film quality deteriorates accordingly. Nevertheless, when the imposed pressure is too high, the PDMS mold may be distorted, pressing abundant photopolymer solution into the cavities. As soon as the pressure is removed, the rubbery soft may restore its geometry and push out the overflowing liquid photopolymer. Replicated film quality is thus decreased.

Figure 5B displays the impact of the table’s shifting speed on the reproducibility of the nanofeatures. The SEM images show that the quality of reproduced nanofeatures lessens as the moving speed is increased. This can be explained by the fact that a pressure is enforced on the glass substrate opposed to the PDMS mold during roller imprinting for certain period in order to press the photopolymer mixture to flow into the nanocavities for nanofeature conformity. As the table is shifted too fast, the photopolymer has no potent time to entirely fulfill into the cavities. Replicated nanofeatures thus deteriorate.

The effect of UV radiation doses on imprinted polymer film quality was also assessed. The microimages in Figure 5C indicate that the duplicability of the imprinted nanofeatures raises as the UV dose is increased. In roller imprinting, after the binary nanosphere array is created on the substrate, the nanofeatures demand UV irradiation to photocure the polymeric mixture into solid. However, as the employed dose of UV irradiation is too low, the replicated nanosphere array may not take in sufficient energy to photocure the polymers. Consequently, the liquid mixture slumps and the duplicability reduces.

**Figure 6:**
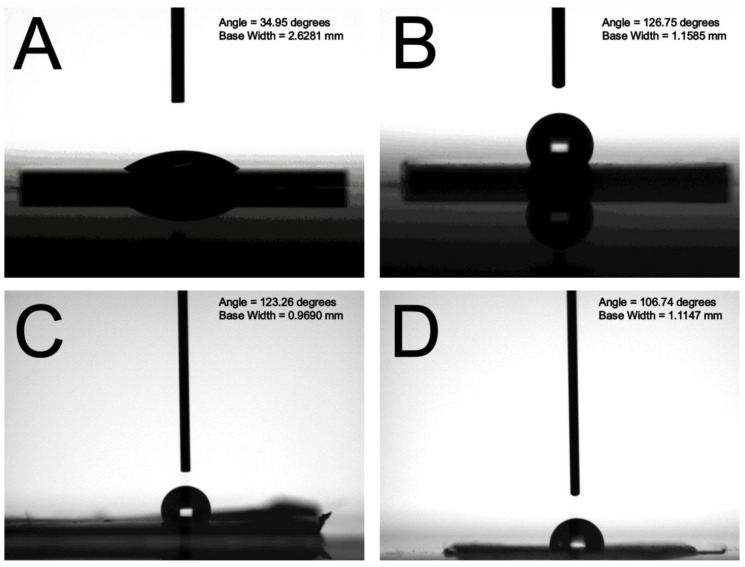
Water contact angles of (**A**) silicon substrate, (**B**) self-assembled binary nanocolloid array, (**C**) replicated soft mold, (**D**) imprinted nanofeatured film.

By employing the appropriate processing parameters, polymeric films with 900 nm/300 nm binary nanosphere array could be satisfactorily prepared (Figure 3C and Figure 4C). The angles of water contact for the self-assembled nanocolloid arrays, duplicated PDMS molds, and imprinted nanostructured films were measured. The measured results in Figure 6 illustrate that the water contact angles for the silicon substrate, assembled binary nanosphere array, replicated soft mold, and imprinted nanofeatured film were 34.95°, 126.75°, 123.26°, and 106.74°, respectively. Imprinted binary nanofeatured films exhibited the expected highly hydrophobic characteristic.

**Figure 7:**
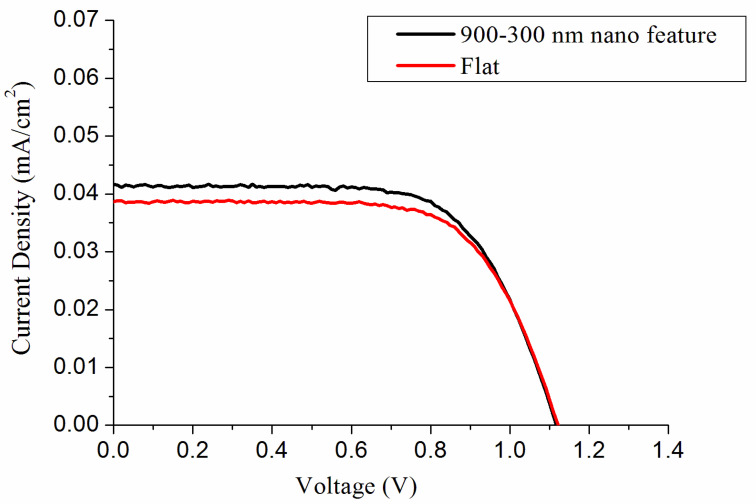
Effect of nanofeatured films on the current-voltage (I-V) characteristics of solar cell.

Finally, to simply demonstrate the capability of imprinted binary nanofeatured films, photocurrent-voltage tests were carried out, utilizing a lab-made device that consists of polycrystalline silicon panel, Xenon lamp that possesses a power supply of 2400 mV (Titan Electro-Optics, Taipei, Taiwan), and simulation code. The distance between the light source and solar cell was maintained at 120 mm, and all measurements were carried out at 25 °C. Figure 7 illustrates the estimated current-voltage profiles of solar cell covered with flat film and nanofeatured film on top of it. Table 2 shows the measured open circuit voltage (Voc), fill factor (FF), short circuit current (Isc), and efficiency of energy transformation (Eff). Binary nanofeatured films demonstrated greater energy transformation efficiency (6.50%) than flat films (5.38%). The Isc and FF increased, mainly due to the fact that the cell absorbs more light because of the nanostructure. Meanwhile, the light captured by the integrated cell/flat film and cell/nanofeatured film systems might be slightly different during measurement, which in turn led to tiny variation of the Voc values. Additionally, the binary nanofeatured films developed in this work exhibited superior energy transformation efficiency to unitary nanofeatured films of either 300 nm (6.02%) or 900 nm (5.96%) [21]. With a binary nanofeature, the surface absorbs most of the incident light, thus reducing reflection, especially in 300–1000 nm wavelength regime [22,23,24]. The binary nanotextured surface also increases the path length of light as it travels through the cell, which in turn enhances energy transformation [25]. Furthermore, compared to unitary structure, hybrid structures are beneficial for absorbing the infrared spectrum of solar radiation, from which a solar cell can absorb more thermal energy [26]. The binary featured surface may also trap the weakly absorbed light reflected from the back surface by total internal reflection at the front surface/air interface. All these promote the efficiency of the solar cell accordingly. Finally, the experimental results in this work suggested that the binary nanostructured films can effectively enhance the energy transformation efficiency of solar cells, which further testified the efficacy of nanosphere lithography and UV roller imprinting for the fabrication of binary nanofeatured surfaces.

## 4. Conclusions

This paper prepared binary nanofeatured films using nanocolloid lithography and ultraviolet roller imprinting. A lab-developed PDMS mold roller imprinting apparatus prepared with UV radiation capability was adopted. The impacts of distinct imprinting variables on the duplicability of binary nanofeatures were investigated. By utilizing the suitable processing parameters, polymeric films with binary 900 nm/300 nm nanosphere arrays can be satisfactorily manufactured. The results in this work show that UV roller imprinting can offer an easy yet potent method to fabricate binary nanostructured film at an ambient temperature with low pressures. This will offer important merits with regard to a minimized fabrication time and improved product quality.

## Figures and Tables

**Table 1:** The operation conditions utilized for spin coating of binary 900 nm/300 nm nanosphere arrays.

Step	Nanosphere Size (nm)	DI Water:Ethanol	Surfactant:PS Sphere	Dispersant (%)	Spin Speed (Spin Time) rpm (s)
One	900	1:1	1:2	5	500 (30) 1500 (30) 2000 (60)
Two	300			10	3000 (30)

**Table 2:** I-V properties of solar cells with flat film and binary nanofeatured film.

Solar Cell	V_max_ (V)	I_max_(mA/cm^2^)	V_OC_ (V)	I_SC_ (mA/cm^2^)	FF (%)	Eff (%)
Flat film	0.69	1.128	0.710	1.323	0.57	5.38
900/300 nm nanofeature	0.62	1.008	0.741	1.396	0.63	6.50

## Data Availability

Data is contained within the article.
